# Automating Misrecognition: The Case of Disability

**DOI:** 10.1007/s11673-025-10462-3

**Published:** 2025-06-03

**Authors:** Jackie Leach Scully, Georgia van Toorn, Sandra Gendera

**Affiliations:** 1 Disability Innovation Institute, UNSW Sydney, Goodsell Building, Kensington, NSW 2052 Australia; 2 School of Social Sciences, UNSW Sydney, Morven Brown Building, Kensington, NSW 2052 Australia; 3 Social Policy Research Centre, UNSW Sydney, Goodsell Building, Kensington, NSW 2052 Australia

**Keywords:** Artificial intelligence, Automated decision-making, Disability, Discrimination, Recognition, Epistemic justice, Human in the loop

## Abstract

Over the last decade, bioethics has begun to address the ethical issues emerging as artificial intelligence (AI) and associated technological processes such as automated decision-making (ADM) become part of healthcare and research. Recent work on justice in AI demonstrates that supposedly neutral AI systems can perpetuate the marginalization of various communities. But so far, there has been little exploration of the interaction of AI and disability. In this empirically based project, we have explored the implications of ADM in the lives of people with disability in Australia. This paper focuses on a point that was consistently raised in discussion by disabled participants but is rarely encountered in the AI ethics literature, especially in relation to disability: the problem of automated systems’ failures of recognition.

Over the last decade, bioethics has begun to address the ethical questions emerging as artificial intelligence (AI) and associated technological processes, such as machine learning (ML) and automated decision making (ADM), become part of healthcare and research. These technologies of complex data processing have ramified through our social lives in profound but often subtle ways. Initially, the ethical discussion centred on the difficulties of ensuring accountability and transparency for complex AI systems, with a more recent focus on issues of bias in the collection and handling of data in AI. The very possibility of bias in AI technologies was neglected for a long time, because of the underlying assumption that computerized algorithms will necessarily be free from the distortions of human decision-making. Recent work on fairness and justice, however, has raised awareness that AI systems can perpetuate biases in various ways. These include (i) the composition of the data entered into the system; (ii) the action of algorithms on the input data; and (iii) how these algorithmic outputs are applied in real-life situations to reproduce existing social inequalities (Mitchell, et al. 2021; Leavy, O’Sullivan, and Siapera 2020; Akter, et al. 2021). Several notable studies have highlighted AI systems’ potential role in perpetuating racism (Noble 2018; Benjamin 2019) and discrimination by class/economic status (Eubanks 2018), with a further recent turn towards the examination of algorithmic sexism (Waelen and Wieczorek 2022). But so far, there has been little exploration of the interaction between AI and disability (Whittaker, et al. 2019).

In an empirical project based within the Australian Centre for Excellence in Automated Decision-Making and Society, we have been exploring the implications of ADM in the lives of people with disability in Australia. An earlier publication (van Toorn and Scully 2023) reported on the case study of the National Disability Insurance Scheme (NDIS), which was scheduled in 2021 to be revolutionized through the integration of algorithmic social profiling into its support planning methods. That study drew on (i) interviews with key informants from disability activism and advocacy networks and officials involved in the independent assessment trials, and (ii) public submissions and Hansard transcripts of evidence to a 2021 parliamentary inquiry into these assessments. One observation made in the course of this study was that disabled informants felt that datafication—the translation of embodied difference into digital data—could not adequately represent the complex realities of disabled lives. To explore this and other points further, a second piece of research aimed to probe in greater depth how non-expert members of the public, both disabled and nondisabled, identified problematic areas in the implementation of ADM in a wider disability context.

We used the method of dialogue groups (DGs), which was devised by one author (JLS) and has been used extensively in research to explore participants’ ethical evaluations of new technologies (see e.g., Banks, et al. 2006; Carter, et al. 2023; Wiley, et al. 2024; Scully, et al. 2017). Dialogue groups are a form of discussion group, in which six to eight participants are provided with some basic but accurate information on the relevant technology. Participants were then presented with fictional scenarios of different situations in which ADM is implemented, designed to illustrate selected ethically troubling aspects of the technology.

A facilitator guided the sequential modification of the scenario as participants moved through the discussion, to pinpoint the features that prompted any shifts in perspective or ethical re-evaluations. This process enables participants both to explore the complexities of the technology and to identify the associated moral values and principles that inform their views. Using a fictional scenario ensures that the discussion moves away from the technical aspects, where participants might have limited understanding, and towards a wider ethical viewpoint.

We held a total of eight DGs: two groups of three experts in either ADM or disability policy (not discussed further here), four groups of six people who self-identified as disabled, and two groups of six members of the general public who did not describe themselves as disabled. In each group, we used one or more of four different scenarios. The first involved the automated surveillance of an elderly person with dementia, the second the computerized assessment of eligibility for disability support, the third was about using AI tools to evaluate applicants for a job, and the fourth focused on an automated assessment of the need for child welfare intervention. Each of the DGs lasted for about ninety minutes and were facilitated by one author while a second was responsible for audio-recording the discussions and taking notes.

Recruitment was managed by Farron Research Group. All participants provided written informed consent to take part and received a $150 e-Gift voucher via Farron. Research Ethics approval was granted by the UNSW Human Research Ethics Committee (HC220370).

For the analysis, one author (SG) first read through and summarized the transcripts. The analysis was thematic, with all three authors independently identifying themes and subthemes from the transcripts and summary, and subsequently meeting to agree on key themes and resolve any differences. Given the small number of transcripts no data management software was used. All quotations used in this paper have been de-identified using pseudonyms instead of participants’ names or other identifying features, including the nature of their disability.

Our participants identified several problematic aspects in each of the scenarios that could be related to different ethical issues associated with the use of ADM. In this paper we focus on one point that was consistently raised by all four groups of people with disability but on only one occasion in the non-disabled groups and that is rarely discussed in the AI ethics literature: the problem of automated systems’ failures of recognition. The remainder of this paper considers this point.

## Recognition and Misrecognition

Recognition describes an engagement with identity—recognizing another person, animal, place, or situation as who or what they are or claim to be. Political philosophy’s examination of recognition has attended to the claims of particular cultural groups for acknowledgement of their distinctive identities, histories, and needs, particularly but not solely as a prerequisite for the just allocation of resources and the redress of past harms (Young 1990; Fraser 1995; Taylor 1992). It also considers the impact of misrecognition, defined by Fraser and Honneth (2003, 134) as “the withdrawal of social recognition, in the phenomena of humiliation and disrespect” and by Giladi, et al. (2018, 145) as what happens when “the recognition order of a society acknowledges the subjectivity of a group or minority, but, incorrectly, does not afford that particular subjectivity the same level of respect and value as that of the majority.” By contrast, philosophical bioethics (including AI ethics) has generally shown less interest in the moral significance of identity, except in terms of the consequences of the social marginalization of particular groups for their health and well-being or the psychological impact of the denial of identities on moral agency (see e.g. Eubanks 2018; Lindemann Nelson 2001).

In their discussions of the DG scenarios, participants with disability related the fictional scenarios of autonomous systems to situations in their own encounters with social services and healthcare in which they felt they had experienced harm due to the lack of recognition. For these participants, AI systems lend themselves to three distinct forms of failed recognition or misrecognition, with none of these forms being a straightforward denial of the existence of disabled people as a group. The first failure concerns the diversity of ways in which disability can be experienced; the second is the epistemic failure to acknowledge the particular kinds of knowledge held by people with disability; and the third is an ontological failure in the encounter between disabled people and the automated system. We will discuss these in turn.

## Recognizing the Diversity of Disability Experience

Artificial intelligence is dependent on the algorithmic processing of data, which in turn relies on datafication: the transformation of phenomena and human experience into the computer legible “language of numbers” (Jasanoff 2004, 27). In terms of disability, the technology sorts human bodies into categories, often based on the diagnostic categorization of impairments (van Toorn 2024). But, as our participants argued, disability is a contested and nuanced concept, and the experience of disability—the point at which social care and healthcare are expected to operate—is not easily represented by algorithmic quantification. Decades of work in disability studies have demonstrated that the individual’s experience of disablement is determined as much by other mediating factors as by the materiality of the impairment itself. What it is like to live with disability is affected by aspects of personal identity (including sex, gender, class, and culture), as well as by prevailing attitudes towards disability, and the organization of the environment and social institutions. Algorithmic calculations are widely criticized for their relative clumsiness in capturing complex, nuanced, and dynamic aspects of human behaviour, and this was brought out in participants’ concern that gross categorizations miss the real heterogeneity of impairment, its relational and fluid nature, and its embeddedness in social structures. In their own lives they felt that ADM systems manage disability by diagnostic categories that take no account of “what it is like” to experience disability:

You tell the algorithms how you feel … they just receive input, they don’t see or feel the disability … a human is still a better choice in this scenario. [Pauline, Group 7, person with disability]

The failure to recognize the form or impact of a person’s disability can have significant effects on any external evaluation of their quality of life or needs, their rights to health or social care, and ultimately their social inclusion. One of our participants related the fictional ADM system to her own experience of automated assessment, saying:

The complete questionnaire didn’t fit at all … none of these questions fitted. So I would just get very cranky … I felt disregarded. [Sarah, Group 3, person with disability and mother of disabled adult]

For many disabled people in the DG, this systematic erasure of lived experience through failed recognition is one of the most troubling and discriminatory aspects of the algorithmic management of disability.

## Recognizing Disabled People’s Knowledge

Several participants described encounters with social services or healthcare in which the particularity of their detailed knowledge of their own disability and needs was disregarded:

… people I know that have dealt with Centrelink, any social services, or whatever it may be, in a scenario like this [with] someone who comes in and goes, “No, it’s not that bad.” Like I’ve dealt with things like depression all my life, anxiety, that sometimes cripples me, and yet I’ve had people at Centrelink go, “No, no, no, there’s nothing wrong with you.” [Simon, Group 7, person with disability]

Porter, Watson, and Pearson (2022, 1165) have described benefits assessments, like those used by the NDIS, as epistemic practices in which “claimants, personal health professionals and commercial assessment providers come together in the production of knowledge about disability.” The disregard of disabled people’s knowledge of disability is a classic example of what has become known as epistemic injustice (van Toorn and Scully 2023). Epistemic injustice results from the facts that, first, social status is accompanied by power over the accumulation and dissemination of knowledge, and second, differences in access to knowledge or control over how it circulates produce a variety of harms. In testimonial injustice (Fricker 2007), prejudice against a group (such as people with disability) results in statements and claims made by members of that group being given less credibility than they would otherwise have.

The dismissal of a person’s own account of their body, its interaction with the world, and its needs and capabilities, is a near-universal experience for disabled people, and is of course not unique to AI or automated systems. However, participants described a particular form of algorithmic injustice that results from the inability of automated systems of data categorization and management to integrate a form of knowledge (in this case, the person’s own knowledge of their disability) that does not fit within the system’s pre-constructed categorie:

Algorithms can do a great job—collecting information, reviewing documents, within set parameters, but when it comes to assessments you need a whole lot more understanding of human conditions. [David, Group 7, person with disability]

In AI, algorithms and the data sets on which they draw become representations of the world, including of disability and impairment, that are programmed into a computerized system. Such algorithmic representations may be inadequate, too rigid, or just wrong, but for both technical and economic reasons are less easily modified than the beliefs held by a human, for example in an assessment:

Even if they [a human assessor] don’t empathize with your condition, they can still put down what they see [unlike an automated system]. [Pauline, Group 7, person with disability]

Epistemic injustice causes harm most obviously because valuable information is not taken into account and the output is therefore less likely to reflect a person’s needs accurately. As Iris Marion Young wrote, “[t]hey are both badly misrepresented and robbed of the means by which to express their perspective” (Young 1990, 59). Epistemic injustice is also fundamentally a form of misrecognition: a person subject to it has not been recognized as a holder of knowledge, and because the capacity to acquire and convey knowledge is something we generally consider essential to human personhood, this is equivalent to the failure to recognize them as persons.

## Ontological Recognition

The third form of failed recognition identified is ontological. Participants suggested that there is a need to feel “seen” or “heard” by another human in a way that acknowledges their existence not just as an equal in a generalized sense but as that *particular person*, with these distinct attributes. Being recognized in one’s particularity has practical consequences because other people’s recognition of you (what they know and believe about you) has a substantial influence on your opportunities and freedom of self-determination. Importantly, it also has consequences for a person’s self-confidence and self-esteem as an individual who belongs and has agency in their world. If, as some theorists argue, a sense of identity is more of a social achievement than an intrinsic property, then without mutual recognition from other human members of their community no individual can fully develop their own personhood.

AI research has seen a recent turn towards an examination of affect, with the aim of understanding better how emotions and social connections can be constructed algorithmically and how people might then respond (DeTogni, et al. 2001; Lee and Norman 2016). But ontological recognition is more than the recognition of certain kinds of affect or patterns of social interaction. Both disabled and nondisabled participants articulated a need for a kind of metaphysical acknowledgement as persons, and were doubtful that an engagement through algorithmic systems would be able to provide this:

…as a human being you do want that recognition that, “OK, your condition or your situation is being acknowledged,” and a computer sitting opposite you is not going to give you that sense, which a human being would. [David, Group 5, person with disability]

## The Need for a Human in the Loop?

All of these concerns about the failures of recognition by automated algorithmic processes raise the question of whether fully autonomous systems, in which there is no human involvement at all, can ever be adequate and acceptable. Data science uses the term “human in the loop,” or HITL, to describe the involvement of people in the computerized gathering, storing, processing, and interpreting of data. The term means slightly different things in the highly diverse contexts of AI design and deployment, but broadly speaking it refers to the view that the end goal of automation may not be the total removal of humans from the system but instead a selective interaction between human and machine, each component bringing their particular strengths to compensate for the other’s weaknesses. Many automated systems designers and AI ethicists have suggested that retaining a human in the loop is essential, particularly in areas where computer error—including errors of mis- or failed recognition—could be catastrophic. The general public, who are uneasy about transferring control over important decisions to algorithms, also tend to be more accepting of systems in which there is still some element of human oversight (Waldman and Martin 2022). As our participants indicated, users believe that the hybrid system retains cognitive and emotional qualities that computers do not have:

[There] should be a human because there are aspects of disability that a computer can’t pick up on … I don’t think [disability assessment] can fully be computerized, and I think you’d feel more comfortable speaking to a human than to a computer. I’d almost feel like I was being taken for a ride to discuss my health issues [with a chatbot]. [Sandra, Group 4, person with disability]

We need a background check from the human element … because mistakes can be made and things can be misjudged. [Malik, Group 3, person with disability]

In this way, the human in the loop is invoked (here, and in AI design and ethics) as a kind of necessary epistemic and moral corrective. There is a belief that human involvement will ensure forms of recognition that machines are incapable of providing.

Yet at the same time, some of our participants questioned the assumption that a human presence would inevitably decrease the risk of misrecognition. In particular, participants with disability who were routinely in contact with arms of the social security, health, or disability support systems, were more likely to have mixed responses when questioned about the importance of a HITL. Their lived experiences include what they described as “biased assessments” and “unfair decisions,” which they traced back specifically to the ignorance or prejudice of human decision makers:

I firmly believe [in] taking away the human element, when it comes to this sort of funding, because I know a lot of people that need disability support don’t get it because the Centrelink worker doesn’t like them or whatever. [Susan, Group 4, person with disability]

In consequence these participants tended to be much more positive about the idea that ADM and related technologies might replace human decision-making altogether. Technology was perceived here as counteracting the potential for ableist stigmatization and discrimination that arises in situations of failed recognition or misrecognition involving humans:

…a computer isn’t going to hopefully judge the person by their appearance, by that three-second rule, by their voice. They’re going to—it’s not going to be biased based on that. It’s going to be fair. This [technology and AI] rules out all of that. So, people will get better care, health care, in the end. [Paula, Group 3, person with disability]

It can’t be any worse [than dealing with a human assessor]. [Simon, Group 7, person with disability]

Other authors have also cautioned against the assumption that having a human in the loop necessarily answers concerns about autonomous decision-making’s lack of empathy or “humanity” (Green and Chen 2019). Krügel, et al. (2023) note, for example, that experiments in which the algorithm makes the decision and the human serves as a corrective sometimes lead to the human turning into a “partner in crime” as they describe it, exploiting a decision made by the algorithm to express their own personal prejudices and bias. Studies of automation bias also suggest that even with a HITL, people often defer their decision-making to automation without scrutiny (Alon-Barkat and Busuioc 2023). In other words, we should be cautious before concluding that human involvement in an ADM system is essential for authentic recognition: it can help reduce the risk of some forms, but to avoid the misrecognition of ableist stereotyping and discrimination requires other safeguards.

## Concluding Discussion

Despite the current high level of activity in the ethics of AI and associated technologies many areas remain under-explored. We argue that the increasing datafication of human life is raising new questions about how recognition is built into ADM systems and what just recognition—or recognition as a matter of justice—means to people for whom such decisions carry significant consequences.

It is worth noting that we also held four DGs of people *without* disability: two where participants had professional expertise in ADM and disability (and were given a more technical scenario), and two groups of non-specialist members of the public. While many of the general concerns about AI were common to all DGs, the issues of misrecognition discussed in the present paper were not prominent in any of the nondisabled groups (there was one mention of the difficulty of capturing disability’s diversity and one of the need to feel recognized by a human). By contrast, people with disability insisted that recognition of their distinctive experiences and knowledge, within the material, economic, social, and relational contexts that shape their lives, is a key criterion for a fair and just system, irrespective of the technology involved. Dencik, et al. (2022, 129) describe datafication as actively generating “forms of subjectification on alien terms in relation to existing conditions, particularly among marginalized groups.” Machine-mediated misrecognition can lead to a sense of alienation and disempowerment, as people’s identities are filtered through the narrow lenses of data-driven systems.

Given most participants’ level of knowledge about AI and ADM, it was not possible to use the discussions to tease out more precisely the most likely source of the failures of recognition, i.e. to differentiate between problems due to biases in the datasets on which algorithms are trained and problems inherent in the design of the algorithm itself. More clarity here will be essential to efforts to address these issues, especially as the rapidly increasing sophistication of AI systems opens up new possibilities of nuance and flexibility. At the same time, greater sophistication is likely to exacerbate the well known “black box” problem in which more complex AI systems become increasingly opaque to external scrutiny.

The majority of both disabled and nondisabled participants in this study preferred a mixed technological and human solution, combined with strengthening of legislation and governance, as a route to achieving fairer system interactions and outcomes for people. At the same time, some of the disabled participants argued that removing the “human” decision element in favour of automation would improve the fairness of social and health systems. We suspect that this reflects their own, damaging experience with existing, human-based systems that are designed, and therefore work best, for the majority population and not for marginalized groups such as people with disability. And irrespective of their conclusion, the criteria they used to evaluate the benefits of a human in the loop were about recognition. Both of these are important points to factor into the future design of inclusive, trustworthy, and just ADM.

## Data Availability

The data that support the findings of this study are not publicly available to protect the confidentiality of the participants. The data are, however, available from the authors upon reasonable request.

## References

[CR1] Akter S., G. McCarthy, S. Sajib, et al. 2021. Algorithmic bias in data-driven innovation in the age of AI*. International Journal of Information Management* 60: 102387.

[CR2] Alon-Barkat S., and M. Busuioc. 2023. Human–AI interactions in public sector decision making: “Automation bias” and “selective adherence” to algorithmic advice. *Journal of Public Administration Research and Theory* 33(1): 153–169.

[CR3] Benjamin, R. 2019. *Race after technology: Abolitionist tools for the New Jim Code*. Medford, MA: Polity.

[CR4] Banks S., J.L. Scully, and T. Shakespeare. 2006. Ordinary ethics: Lay people’s deliberations on social sex selection. *New Genetics and Society* 25(3): 289–303

[CR5] Carter, S.M., D. Popic, M. Marinovich, L. Carolan, and N. Houssami. 2024. Women’s views on using artificial intelligence in breast cancer screening: A review and qualitative study to guide breast screening services. *The Breast* 77: 10.1016/j.breast.2024.103783. 10.1016/j.breast.2024.103783 PMC11362777 39111200

[CR6] Denci, L. 2022. Data and social justice. In *Data justice*, edited by L. Dencik, A. Hintz., J. Redden, and E. Treré, 123–138. London: Sage

[CR7] De Togni, G., S. Erikainen, S. Chan, and S. Cunningham-Burley. 2021. What makes AI “intelligent” and “caring”? Exploring affect and relationality across three sites of intelligence and care. *Social Science & Medicine* 277: 113874. 33901725 10.1016/j.socscimed.2021.113874 PMC8135128

[CR8] Eubanks, V. 2018. *Automating inequality: How high-tech tools profile, police, and punish the poor.* New York: Picador, St Martin’s Press.

[CR9] Fraser, N., and A. Honneth. 2003. *Redistribution or recognition? A political-philosophical exchange*. London: Verso.

[CR10] Fraser, N. 1995. Recognition or redistribution? A critical reading of Iris Young’s Justice and the politics of difference. *Journal of Political Philosophy* 3(2): 166–180.

[CR11] Fricker, M. 2007. *Epistemic injustice: Power and the ethics of knowing*. Oxford: Oxford University Press.

[CR12] Giladi, P. 2018. Epistemic injustice: A role for recognition? *Philosophy & Social Criticism* 44(2): 141–158.

[CR13] Green, B., and Y. Chen. 2019. The principles and limits of algorithm-in-the-loop decision making. *Proceedings of the ACM on Human-Computer Interaction* 3(CSCW): 50: 1–50: 24.

[CR14] Jasanoff, S. 2004. Ordering knowledge, ordering society. In *States of knowledge: The co-production of science and the social order*, edited by S. Jasanoff, 13–45. London: Routledge.

[CR15] Krügel, S., A. Ostermaier, and M. Uhl. 2023. Algorithms as partners in crime: A lesson in ethics by design. *Computers in Human Behavior* 138: 107483.

[CR16] Leavy, S., B. O’Sullivan, and E. Siapera. 2020. Data, power and bias in Artificial Intelligence. *AI for Social Good Workshop*.

[CR17] Lee, W., and M.D. Norman. 2016. Affective computing as complex systems science. *Procedia Computer Science* 95: 18–23.

[CR18] Lindemann Nelson, H. 2001. *Damaged identities, narrative repair*. Ithaca: Cornell University Press.

[CR19] Mitchell, S., E. Potash, S. Barocas, A. D’Amour, and K. Lum. 2021. Algorithmic fairness: Choices, assumptions, and definitions. *Annual Review of Statistics and Its Application* 8(1): 141–163.

[CR20] Noble, S.U. 2018. *Algorithms of oppression: How search engines reinforce racism*. New York: New York University Press. 10.1126/science.abm5861 34709921

[CR21] Porter, T., N. Watson, and C. Pearson. 2023. Epistemic sabotage: The production and disqualification of evidence in disability benefit claims. *Sociology of Health & Illness* 45(6): 1164–1186. 36529900 10.1111/1467-9566.13593

[CR22] Scully, J.L., Banks, S., Song, R., and J. Haq. 2017. Experiences of faith group members using new reproductive and genetic technologies: A qualitative interview study. *Human Fertility* 20(1): 22–29. 27841038 10.1080/14647273.2016.1243816

[CR23] Taylor, C. 1992. The politics of recognition. In *Multiculturalism and the Politics of Recognition*, edited by A. Gutmann, 25–74. Princeton: Princeton University Press.

[CR24] van Toorn, G., and J.L. Scully. 2023. Unveiling algorithmic power: Exploring the impact of automated systems on disabled people’s engagement with social services. *Disability & Society* 39(11): 3004–3029.

[CR25] van Toorn, G. 2024. Automating the welfare state. In *The Routledge Handbook of the Political Economy of Health and Healthcare*, edited by D. Primrose, R.D. Loeppky, and R. Chang, 259–270. London: Routledge.

[CR26] Waelen, R., and M. Wieczorek. 2022. The struggle for AI’s recognition: Understanding the normative implications of gender bias in AI with Honneth’s theory of recognition. *Philosophy & Technology* 35: 53.

[CR27] Waldman, A., and K. Martin. 2022. Governing algorithmic decisions: The role of decision importance and governance on perceived legitimacy of algorithmic decisions. *Big Data & Society* 9(1): 10.1177/20539517221100449.

[CR28] Whittaker, M., M. Alper, C.L. Bennett, et al. 2019. Disability, bias, and AI. *AI Now Institute*. https://ainowinstitute.org/wp-content/uploads/2023/04/disabilitybiasai-2019.pdf. Accessed May 23, 2022.

[CR29] Wiley, K., P. Robinson, C. Degeling, P. Ward, J.Leask, and S. Carter. 2023. “Get your own house in order”: Qualitative dialogue groups with nonvaccinating parents on how measles outbreaks in their community should be managed. *Health Expectations* 25(4): 1678–1690. 10.1111/hex.13511 PMC9327825 35548872

[CR30] Young, I.M. 1990. *Justice and the politics of difference*. Princeton, N.J.: Princeton University Press.

